# Intrauterine Transfer of Polyunsaturated Fatty Acids in Mother–Infant Dyads as Analyzed at Time of Delivery

**DOI:** 10.3390/nu13030996

**Published:** 2021-03-19

**Authors:** Vanessa Woodard, Melissa Thoene, Matthew Van Ormer, Maranda Thompson, Corrine Hanson, Sathish Kumar Natarajan, Maheswari Mukherjee, Ana Yuil-Valdes, Tara M. Nordgren, Arzu Ulu, Kristina Harris Jackson, Ann Anderson-Berry

**Affiliations:** 1 Department of Pediatrics, College of Medicine, University of Nebraska Medical Center, Omaha, NE 68198, USA; vanessa.woodard@unmc.edu (V.W.); melissak.thoene@unmc.edu (M.T.); matthew.vanormer@unmc.edu (M.V.O.); maranda.thompson@unmc.edu (M.T.); ana.yuilvaldes@unmc.edu (A.Y.-V.); alanders@unmc.edu (A.A.-B.); 2 Department of Medical Sciences, College of Allied Health Professions, University of Nebraska Medical Center, Omaha, NE 68198, USA; mmukherj@unmc.edu; 3 Department of Nutrition and Health Sciences, College of Education and Human Sciences, University of Nebraska at Lincoln, Lincoln, NE 68583, USA; snatarajan2@unl.edu; 4 Division of Biomedical Sciences, College of Medicine, University of California Riverside, Riverside, CA 92521, USA; tara.nordgren@medsch.ucr.edu (T.M.N.); arzu.ulu@medsch.ucr.edu (A.U.); 5 OmegaQuant Analytics LLC, 5009 W. 12th St., Suite 8, Sioux Falls, SD 57106, USA; kristina@omegaquant.com; 6 Department of Internal Medicine, College of Medicine, University of South Dakota Sanford School of Medicine, Vermillion, SD 57069, USA

**Keywords:** omega-3 fatty acids, PUFA, intrauterine transfer, maternal-fetal health, placenta

## Abstract

Polyunsaturated fatty acids (PUFAs) are essential for fetal development, and intrauterine transfer is the only supply of PUFAs to the fetus. The prevailing theory of gestational nutrient transfer is that certain nutrients (including PUFAs) may have prioritized transport across the placenta. Numerous studies have identified correlations between maternal and infant fatty acid concentrations; however, little is known about what role maternal PUFA status may play in differential intrauterine nutrient transfer. Twenty mother–infant dyads were enrolled at delivery for collection of maternal and umbilical cord blood, and placental tissue samples. Plasma concentrations of PUFAs were assessed using gas chromatography (GC-FID). Intrauterine transfer percentages for each fatty acid were calculated as follows: ((cord blood fatty acid level/maternal blood fatty acid level) × 100). Kruskal–Wallis tests were used to compare transfer percentages between maternal fatty acid tertile groups. A *p*-value < 0.05 was considered significant. There were statistically significant differences in intrauterine transfer percentages of arachidonic acid (AA) (64% vs. 65% vs. 45%, *p* = 0.02), eicosapentaenoic acid (EPA) (41% vs. 19% vs. 17%, *p* = 0.03), and total fatty acids (TFA) (27% vs. 26% vs. 20%, *p* = 0.05) between maternal plasma fatty acid tertiles. Intrauterine transfer percentages of AA, EPA, and TFA were highest in the lowest tertile of respective maternal fatty acid concentration. These findings may indicate that fatty acid transfer to the fetus is prioritized during gestation even during periods of maternal nutritional inadequacy.

## 1. Introduction

Polyunsaturated fatty acids (PUFAs) are essential for growth and development during pregnancy, particularly structural growth in the third trimester such as cell membranes, the retina, and the brain. During the perinatal period, PUFAs also aid in the development of a functional immune system by altering the function of regulatory T cells, the growth of fetal neural cells, and signal transduction [1]. While in utero, fetuses rely on transfer of PUFAs through the placenta and umbilical cord; some studies have shown that maternal dietary intake of PUFAs can impact umbilical cord concentrations [1,2].

Although PUFAs have been extensively studied in pregnancy, the dynamics of intrauterine nutrient transfer across the placenta are still not well understood. Transfer of nutrients across the placenta is the single source of nutrition for a growing fetus and, as such, transfer of these nutrients is critical for appropriate fetal development. Compounds that cross the placenta efficiently are small, uncharged molecules, such as oxygen, which depend on rate of blood flow to be able to cross the placenta [3]. Larger charged molecules are often able to cross the placenta with the aid of intraplacental transporters, as is the case with glucose crossing the placenta via glucose transporters (GLUT) proteins [3,4]. PUFAs including linoleic acid (LA), α-linolenic acid (ALA), arachidonic acid (AA), docosapentaenoic acid (DPA), docosahexaenoic acid (DHA), and eicosapentaenoic acid (EPA) are large molecules and do not readily cross the placenta; placental transport of fatty acids is heavily transporter dependent, and the mechanisms that regulate preferential transfer remain poorly understood [5]. Among nutrients that are highly transferred, such as glucose, there does not seem to be a consistent correlation between maternal plasma level and fetal growth [6]. However, micronutrients that may be subject to tighter regulation have shown correlation to fetal growth such as retinol, which is essential during pregnancy to support fetal growth [7]. Placental PUFA transfer appears to rely largely on plasma membrane bound proteins that bind non-esterified fatty acids and transport them across the membrane, with a small number of fatty acids being able to cross the placental membrane via passive diffusion [8]. Since the maternal to fetal gradient of free fatty acids is high, some passive diffusion can occur; however, these fatty acids are processed and esterified prior to release into the fetal circulation [9].

Throughout the course of pregnancy, maternal plasma PUFA concentrations decrease with ultimately higher concentrations of fatty acids in cord blood at delivery than in maternal plasma [10]. For example, increased concentrations of DHA have been found in cord blood compared to maternal plasma concentration, indicating there may be some selectivity for specific fatty acids to more easily transfer across the placenta [4]. In some studies, placental concentrations of DHA and AA have been shown to be up to 4-fold higher in the placental intervillous space than in the maternal plasma, indicating an increase in the transfer of these specific fatty acids [11]. Some studies have been conducted to investigate the mechanics of intrauterine PUFA transfer using radio-labelled fatty acid supplements [12], and others have evaluated direct correlations between maternal and fetal fatty acid levels [13,14,15,16]; however, minimal investigation has been conducted on differential intrauterine PUFA transfer in the context of varied maternal nutrient status. Therefore, the purpose of this study is to quantify the intrauterine transfer percentages of six PUFAs between maternal plasma fatty acid tertile groups.

## 2. Materials and Methods

### 2.1. Subjects

An Institutional Review Board-approved (#112-15-EP, 4/14/15) study at the University of Nebraska Medical Center (Omaha, NE, USA) screened and enrolled twenty mother–infant dyads at the time of delivery. Informed written consent was acquired from the mother (for herself and her infant) prior to study participation. Inclusion criteria included mothers ≥ 19 years of age and delivering at least one live-born infant at term gestational age (≥37 weeks gestation [17]), and exclusion criteria included congenital abnormalities, inborn errors of metabolism, other conditions that may impact the metabolism of fatty acids, and infants deemed wards of the state.

Only mother–infant pairs with both maternal and umbilical cord plasma results were included in this analysis.

### 2.2. Sample Collection

Maternal blood, umbilical cord blood, and placental tissue cross-sections were collected from participants at time of delivery by study personnel, and promptly processed and stored at −80 °C. Target volume for plasma samples was 150 µL of plasma, and placental cross sections had a target volume of 1 g. All samples were protected from heat and light to minimize degradation of unstable molecules.

### 2.3. Fatty Acid Analysis

Plasma fatty acid composition was analyzed by gas chromatography with flame ionization detection (GC-FID) at OmegaQuant Analytics LLC (Sioux Falls, SD, USA). Plasma was transferred to a screw-cap glass vial and boron trifluoride-methanol (BTM) solution (methanol containing 14% boron trifluoride, toluene, methanol; 35:30:35 v/v/v; Sigma-Aldrich, St. Louis, MO, USA) was added. The vial was briefly vortexed and heated in a hot bath at 100 °C for 45 min. After cooling, hexane (Merck KGaA, Darmstadt, Germany) and high-performance liquid chromatography grade water was added. The tubes were recapped, vortexed, and centrifuged to separate layers. An aliquot of the hexane layer was transferred to a GC vial. GC was carried out using a GC-2010 Gas Chromatograph (Shimadzu Corporation, Columbia, MD, USA) equipped with a SP-2560, 100 m fused silica capillary column (0.25 mm internal diameter, 0.2 um film thickness; Supelco, Bellefonte, PA, USA).

Fatty acids were identified by comparison with a standard mixture of fatty acids (GLC OQ-A, NuCheck Prep, Elysian, MN, USA) which was also used to determine individual fatty acid calibration curves. PUFAs of interest include linoleic acid (LA), α-linolenic acid (ALA), arachidonic acid (AA), docosapentaenoic acid (DPA), docosahexaenoic acid (DHA), eicosapentaenoic acid (EPA), and Total Fatty Acids (TFA).

### 2.4. Clinical and Demographic Data

Demographic variables included infant sex and maternal race. Clinical data included delivery mode (vaginal vs. caesarean), birth weight in grams, gestational age in weeks, birth length in centimeters, and birth head circumference in centimeters. We additionally collected maternal pre-pregnancy body mass index (BMI). All data were collected from the electronic medical record at the time of delivery. Maternal dietary intake was quantified by completing the Willet Food Frequency Questionnaire, a quantitative survey that calculates daily nutrient intake based on reported diet over the last year, and is validated in multiple populations including pregnant women [18].

### 2.5. Statistical Analysis

Descriptive statistics were calculated for all variables, including median and interquartile ranges (IQR) for continuous variables and counts and proportions are presented for categorical variables. Intrauterine transfer percentage for each PUFA was calculated as ((umbilical cord blood fatty acid level/maternal blood fatty acid level) × 100). The Kruskal–Wallis test and post-hoc tests were used to compare intrauterine transfer percentages between maternal fatty acid tertiles. IBM SPSS Statistics 25 software (IBM Corp., New York, NY, USA) was used for statistical analyses. A *p*-value < 0.05 was considered statistically significant.

## 3. Results

There were 20 mother–infant dyads included in this study. Demographic data for participants are displayed in Table 1.

The raw concentrations of each measured PUFA in maternal plasma, umbilical cord, and placenta, which were used to calculate transfer percentage, can be found in Table 2, Table 3 and Table 4, respectively. The maternal dietary intake as determined by Willet Food Frequency Questionnaire for each nutrient is provided in Table 5.

There was a statistically significant difference in intrauterine transfer percentages of AA, EPA, and TFA between maternal fatty acid tertiles (Table 6). Transfer percentages of AA, EPA, and TFA were highest in mothers in the lowest tertile of respective fatty acid concentration. For EPA, mothers in the middle and highest tertiles transferred 19.3% and 17.6%, respectively, whereas mothers in the lowest tertile of plasma concentration transferred 41.1% (*p* = 0.03). For AA, mothers in the highest tertile of maternal plasma level transferred 45.2%, while mothers in the middle and lowest tertiles transferred 65.8% and 64.8%, respectively (*p* = 0.02). The TFA transfer pattern is similar to AA; TFA in mothers with the highest tertile of maternal plasma level transferred 19.6%, while mothers in the middle and lowest tertiles transferred 25.7% and 26.9%, respectively (*p*-value = 0.05).

**Figure 1:**
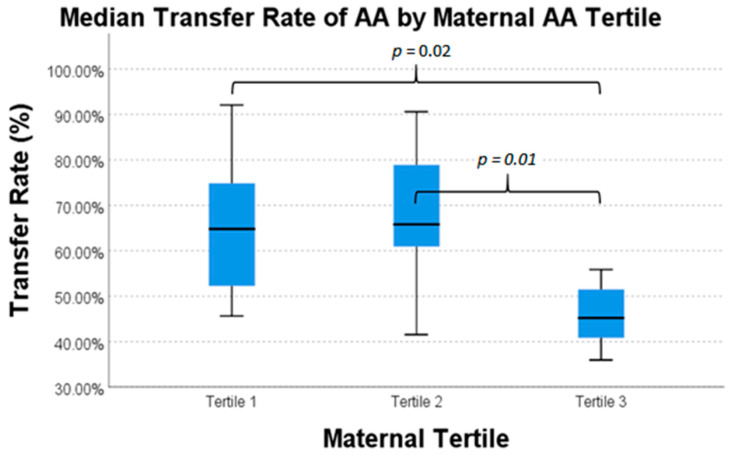
Median transfer percentage of arachidonic acid (AA) across tertiles of maternal plasma AA concentration.

**Figure 2:**
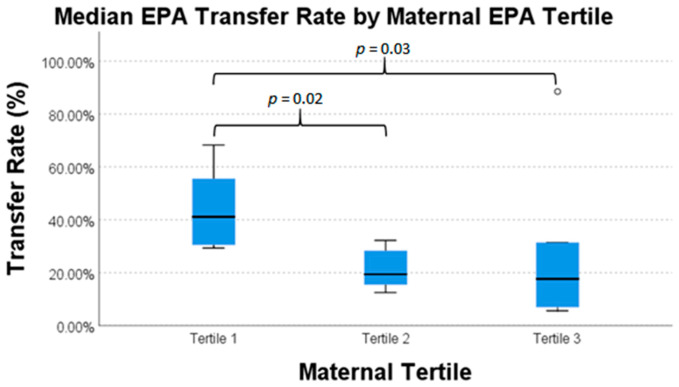
Intrauterine transfer percentage of eicosapentaenoic acid (EPA) across tertiles of maternal plasma concentration.

**Figure 3:**
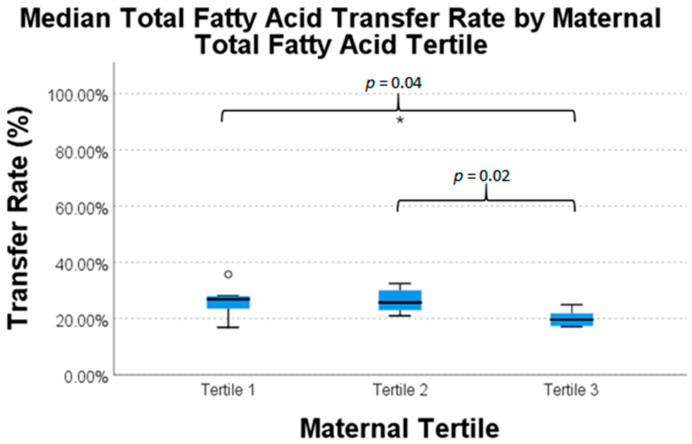
Intrauterine transfer percentage of total fatty acid across tertiles of maternal plasma concentration. Asterisk refers to Tertile 2.

Pairwise comparisons for each nutrient are presented in Figure 1, Figure 2 and Figure 3. These data exhibit a statistically significantly difference for mothers of the highest tertile of AA concentration versus mothers of the middle and lowest tertile of AA concentration (Figure 1). There was also a statistically significant difference in the transfer of EPA in mothers in lowest tertile and the transfer of EPA in mothers in the two highest tertiles (Figure 2). There was again a statistically significant difference between transfer of TFA in mothers of the lowest tertile and mothers of the two highest tertiles (Figure 3). This difference in intrauterine transfer by maternal fatty acid tertile was not seen in DPA, DHA, LA, or ALA.

## 4. Discussion

The findings of this study may indicate that there may be a prioritization of intrauterine transfer of some, but not all, fatty acids to the fetus, particularly in the setting of low maternal nutrient status [11]. We have earlier surveyed the amount of fatty acid intake among multiple population groups, including pregnant women, from 2003 to 2014 using the National Health and Nutrition Examination Survey database and found that pregnant women are well below the recommended dietary intake levels of omega-3 fatty acid intakes [19]. Recommended daily allowance of omega-3 fatty acid dietary intake in pregnant women varies widely, with some organizations recommending up to 650 mg/day with at least 220 mg/day of each DHA and EPA [20]. Failure to achieve these levels could result in latent consequences to the fetus, as PUFAs are not only important for intrauterine development of the retina and brain [4] but also for postnatal visual function and neurodevelopment [21]. The nutritional environment during early pregnancy may even affect fetal gene expression, particularly in the case of PUFA deficiency where there may not be enough PUFAs to reduce inflammation [22]. In this study, we saw that median total omega-3 fatty acid intake reached recommended intake levels for pregnant women, with their average total dietary intake of omega-3 fatty acids 1330 mg, but median intake of DHA was only 60 mg and median intake of EPA was just 10 mg (Table 5). Interestingly, in mothers with the lowest levels of plasma EPA, we identified the highest intrauterine transfer percentages of EPA. These data suggest that certain fatty acids essential to pregnancy may be selectively transferred to the infant, regardless of maternal nutrition status. However, DHA and DPA did not exhibit differential transfer percentages amongst different maternal plasma PUFA tertiles, suggesting that infants could be at risk for lower PUFA status in one of these important omega-3 fatty acids in mothers having lower DHA and DPA intakes or plasma levels. Of note, a previous study evaluating the mechanics of intrauterine fatty acid transfer using radiolabeled fatty acid supplements indicated a significantly higher accretion of DHA in the placental tissue compared to palmitic acid, oleic acid, and linoleic acid [12]—this adds important context to our findings, in that DHA may not have variable transfer from maternal blood to cord blood based on maternal DHA status due to its high uptake in the interposed placental tissue. Taken in conjunction with previous studies indicating poor intake of PUFAs in pregnant women in the United States [19], our results highlight that some, but not all, fatty acids may have increased intrauterine transfer when maternal stores are low. These data may indicate the importance of targeted nutrient supplementation and strategic diet modifications to optimize nutrient availability in women of childbearing age.

These results, which show a potentially increased transfer of AA, may be counter-intuitive given AA’s reputation as a substrate for the production of many pro-inflammatory mediators. However, AA is a PUFA which is essential for many normal human functions, particularly in the nervous system [23]. Additionally, while AA contributes to Type II immune responses, its oxidative metabolites such as downstream P450 metabolites including epoxyeicosatrienoic acids, contribute to the resolution of inflammation [23,24]. Given the prolonged period of development, intrauterine augmentation of AA transfer to the fetus may be beneficial to support critical neonatal development [25,26]. Since AA has a proven benefit to nervous system function, augmentation of intrauterine transfer may be necessary given the longevity and cruciality of fetal nervous system development throughout pregnancy. In contrast to AA, other PUFAs that did not appear to have increased transfer percentages are also essential for fetal development. LA and ALA are considered essential fatty acids that must be obtained from the diet, since they contain molecularly structured bonds that cannot be synthesized by the body [27]. LA and ALA are the building blocks of AA, EPA, DHA. It has been hypothesized that the concentration of AA, EPA, and DHA are markers of placental function [11]. Thus, our findings of increased AA and EPA may point towards the optimal function of the placenta in these healthy pregnancies in terms of metabolism of ALA and LA.

The amount of available free fatty acids for a fetus may have a positive impact on appropriate fetal development. Previous studies have evaluated fatty acid correlations between maternal and infant blood, as well as their relationship with infant outcomes. For instance, Meher et al. [13] assessed differences in maternal and cord fatty acid levels between low birthweight infants and appropriate for gestational age infants, as well as the correlations between maternal and infant erythrocyte fatty acid concentrations [13]. This study observed direct correlations between maternal and cord DHA, AA, and TFA at multiple timepoints during pregnancy. However, our study aims to assess the intrauterine transfer of fatty in the context of disparate maternal nutrient status. Other studies have discussed the dynamics of biomagnification in the context of PUFAs [14,28], and the potential for prioritized nutrient transfer regardless of low available maternal substrate. Another potential implication of increased PUFA substrate is the anti-inflammatory properties of PUFAs, especially omega-3 fatty acids [29]. Increased PUFA transfer across the placenta over the course of pregnancy has been hypothesized to prepare a fetus for the oxidative stresses of extrauterine life [29]. The downstream metabolites of omega-3 fatty acids have been shown to promote the resolution of inflammatory cascades [30]. Since intrauterine transfer of certain fatty acids seems to be prioritized, it may be possible that this is due to the increased need for anti-inflammatory substrate, particularly in the first few months of life as infants are susceptible to infection prior to the full development of an infantile immune system [31,32]. The results of this study suggest a fetal requirement for a minimal concentration of PUFAs which may potentially have positive implications for fetal development, inflammation resolution, and improved neurodevelopmental outcomes.

### Strengths and Limitations

This study population included mother–infant dyads of term pregnancies, which limits the confounding role that placental abnormality, morbidities of prematurity, and differences in placental development may have on intrauterine nutrient transfer. However, a very small sample size limits our ability to make strong claims that these results are generalizable to a broader population, as well as adjusting our findings for potential confounding effects. Specifically, the significant difference in TFA transfer percentage may be the product of the significantly different transfer percentages of two constituent parts (AA and EPA). However, we were unable to assess this possibility statistically given our small sample size. Further analyses of PUFA intrauterine transfer percentages in a larger cohort of mother–infant dyads will be necessary to determine the validity and reproducibility of the results presented in this manuscript. An additional limitation of this study is that our study evaluated fatty acid levels in maternal and infant plasma rather than erythrocyte composition. There is some discussion about which methodology is most appropriate to assess nutrient status, particularly over longer periods of time [33]. In future studies, we plan to evaluate fatty acid plasma concentration and erythrocyte composition in parallel to address this potential limitation. Finally, the possibility of a selection bias in that all subjects of this study were pregnancies that were carried to full-term. This selection bias could discount the possibility that fetuses who reached full term as those who are able to receive enough fatty acid via intrauterine transfer and potentially mothers of lower nutritional status are more likely to have preterm infants and were thus not included in this study. Nonetheless, we report data answering to an important question of differential fatty acid transfer in the setting of pregnancy, with indications that fetal nutrient delivery of certain fatty acids is prioritized even in deplete settings.

## 5. Conclusions

Our data in the present study indicate that intrauterine transfer of AA, EPA, and TFA is higher in mothers in the lowest tertiles of their respective plasma fatty acid concentrations. This difference was not seen in the transfer of DPA, DHA, LA, or ALA. These results imply that there is a minimum transfer percentage of specific nutrients required for appropriate fetal development, regardless of maternal nutrition status. Given the importance of PUFAs in fetal development, these results may further support the importance of achieving the recommended dietary levels of PUFAs in pregnant women and also targeting specific maternal PUFA deficiencies via supplementation in cases where intrauterine transfer percentages do not differ significantly based on maternal serum levels (e.g., DHA and DPA).

## Figures and Tables

**Table 1:** Demographic Data of Mother–Infant Dyads.

	Median	IQR	Min	Max
Infant CGA (weeks)	39.45	39.0–40.2	37.4	41.0
Infant birthweight (g)	3467	3172.5–3758.0	2955	4893
Infant birth length (cm)	50.50	48.3–50.8	45.7	53.3
Infant birth head circumference (cm)	34.60	33.0–35.6	32.4	36.8
Maternal pre-pregnancy BMI (kg/m^2^)	24.7	21.2–28.9	18.2	43.9
	Count (*n*)	Percentage (%)		
Infant sex:				
Male	11	55.0		
Female	9	45.0		
Maternal race:				
White	10	50.0		
Black/African American	4	20.0		
Hispanic/Latino	3	15.0		
Asian/Pacific Islander	1	5.0		
Native American/Alaskan	0	0.0		
Other/Unknown	2	10.0		
Delivery mode:				
Vaginal	18	90.0		
Caesarean	2	10.0		

CGA = Corrected Gestational Age, BMI = Body-Mass Index.

**Table 2:** Concentrations of PUFAs in Maternal Plasma.

	Median	IQR	33rd %ile	67th %ile	Min	Max
Linoleic Acid (µg/mL)	1482.6	1322.0–1618.9	1342.4	1584.5	973.9	2122.3
α-Linolenic Acid (µg/mL)	35.6	28.5–39.4	33.8	39.4	24.2	52.5
Arachidonic Acid (µg/mL)	272.7	202.8–312.6	224.21	289.2	183.5	417.6
Eicosapentaenoic Acid (µg/mL)	6.0	4.0–9.3	5.0	7.2	2.0	18.5
Docosapentaenoic-n3 Acid (µg/mL)	10.1	9.0–12.0	9.2	12.0	7.0	20.8
Docosahexaenoic Acid (µg/mL)	72.7	59.4–81.7	62.4	80.4	37.6	161.3
Total Fatty Acids (µg/mL)	4794.0	4062.2–5208.2	4601.1	5093.7	3423.8	7138.1

%ile = percentile

**Table 3:** Concentrations of PUFAs in Umbilical Cord Plasma.

	Median	IQR	Min	Max
Linoleic Acid (µg/mL)	136.8	101.0–184.7	89.6	1342.6
α-Linolenic Acid (µg/mL)	2.5	1.2–3.1	0.7	40.0
Arachidonic Acid (µg/mL)	162.6	125.8–181.7	89.4	251.6
Eicosapentaenoic Acid (µg/mL)	1.2	0.9–2.5	0.7	8.4
Docosapentaenoic-n3 Acid (µg/mL)	2.4	1.7–3.5	0.7	10.0
Docosahexaenoic Acid (µg/mL)	36.7	30.1–46.5	19.2	83.3
Total Fatty Acids (µg/mL)	1155.5	964.5–1341.8	721.2	4621.2

IQR = Interquartile Range

**Table 4:** Concentrations of PUFAs in Placenta.

	Median	IQR	Min	Max
Linoleic Acid (µg/mg)	1.11	1.0–1.3	0.72	1.56
α-Linolenic Acid (µg/mg)	0.01	0.009–0.012	0.01	0.02
Arachidonic Acid (µg/mg)	2.09	1.8–2.3	1.28	2.51
Eicosapentaenoic Acid (µg/mg)	0.01	0.005–0.010	0.00	0.02
Docosapentaenoic-n3 Acid (µg/mg)	0.07	0.05–0.08	0.03	0.12
Docosahexaenoic Acid (µg/mg)	0.32	0.26–0.44	0.18	0.64
Total Fatty Acids (µg/mg)	9.97	8.63–10.72	6.82	12.46

IQR = Interquartile Range

**Table 5:** Maternal PUFA Dietary Intake.

	Median	IQR	Min	Max
Linoleic Acid (g)	9.42	8.2–14.2	1.8	18.7
α-Linolenic Acid (g)	1.19	0.9–1.4	0.2	1.9
Arachidonic Acid (g)	0.12	0.1–0.2	0.0	0.3
Eicosapentaenoic Acid (g)	0.01	0–0.05	0.0	0.4
Docosapentaenoic-n3 Acid (g)	0.02	0.01–0.02	0.0	0.1
Docosahexaenoic Acid (g)	0.06	0.03–0.12	0.0	0.4
Total Omega-3 Acid (g)	1.33	0.9–1.6	0.22	2.01

IQR = Interquartile Range

**Table 6:** Kruskal–Wallis Test of Intrauterine Transfer Percentage by Maternal Plasma Level Tertile.

	*n*	Tertile 1 (Lowest)	Tertile 2	Tertile 3 (Highest)	*p*-Value
Linoleic Acid	20	10.3%	9.2%	9.8%	0.86
α-linolenic Acid	20	4.8%	7.0%	6.8%	0.68
Arachidonic Acid	20	64.8%	65.8%	45.2%	0.02 *
Docosapentaenoic Acid	20	31.0%	20.3%	20.1%	0.68
Docosahexaenoic Acid	20	57.8%	52.4%	45.9%	0.60
Eicosapentaenoic Acid	20	41.1%	19.3%	17.7%	0.03 *
Total Fatty Acids	20	26.9%	25.7%	19.6%	0.05 *

* indicates *p*-value ≤ 0.05

## Data Availability

The data presented in this study are available on request from the corresponding author. The data are not publicly available due to protecting patient confidentiality.
